# Case report: A case of renal arcuate vein thrombosis successfully treated with direct oral anticoagulants

**DOI:** 10.3389/fmed.2023.1092815

**Published:** 2023-06-20

**Authors:** Mahsa Torabi Jahromi, Jamshid Roozbeh, Fatemeh Masjedi, Sahand Mohammadzadeh, Seyed Sajjad Tabei, Maryam Shafiee, Nakisa Rasaei

**Affiliations:** ^1^Shiraz Nephro-Urology Research Center, Shiraz University of Medical Sciences, Shiraz, Iran; ^2^Department of Pathology, School of Medicine, Shiraz University of Medical Science, Shiraz, Iran; ^3^Division of Urology, Department of Surgery, University of Cincinnati College of Medicine, Cincinnati, OH, United States

**Keywords:** renal arcuate vein thrombosis, direct oral anticoagulants, acute kidney injury, thromboembolism, Apixaban

## Abstract

A rare case of a 35 years old woman presented with renal arcuate vein thrombosis (RAVT) and acute kidney injury (AKI) following upper respiratory tract symptoms and toxic substance ingestion. Histopathological evaluation of the patient's kidney tissue indicated a rare venous thrombosis in the renal arcuate veins. Anticoagulation with Apixaban, a direct oral anticoagulant (DOAC), was commenced, and the patient's symptoms resolved during the hospital stay. Hitherto, a limited number of studies have shown the concurrent presentation of RAVT and overt AKI in patients following ingestion of nephrotoxic agents. Further studies are necessary to elucidate the etiology, clinical presentation, and treatment of RAVT. We suggest that Apixaban be studied as a suitable alternative to conventionally used anti-coagulants such as Warfarin in patients who lack access to optimal health care facilities.

## Background

Thromboembolic events in the renal veins are rare and underdiagnosed status that can cause acute kidney injury (AKI) with life-threatening related conditions (1).

Due to the possible asymptomatic emergence and spontaneous resolution in some cases, renal vein thromboses (RVT) are often challenging to diagnose and be discovered incidentally by imaging studies (2–4). Therefore, the exact prevalence of RVT may be underestimated in the general population. However, special populations, such as renal transplant patients, present a prevalence rate of 0.1 to 6% (2, 3). Epidemiological studies on other disease groups, such as those afflicted with nephrotic syndrome and membranous nephropathy, have also shown a 5 to 60% prevalence rate (5, 6).

The etiology of RVT is no different from other forms of venous thrombosis and must be evaluated in the context of Virchow's triad (7). While states of dehydration are one of the most prevalent causes of RVT development in neonates, and RVT most commonly occurs secondary to nephrotic syndrome and renal transplantation in children (4), the etiology of RVT differs in adults. Glomerular pathologies such as nephrotic syndrome, hyper-coagulability, membranous glomerulonephritis, and non-glomerular entities such as neoplasia, rheumatologic disorders, prior abdominal surgeries, trauma, and oral contraceptive use are known to play a role in RVT development in adults (8).

The most common clinical features of RVT that should be considered include flank pain, gross hematuria, nausea/vomiting, asterixis, and subsequent anemia (9).

An established cause of acute kidney failure is venous thrombosis of the extrarenal veins. While intrarenal venous thrombosis isolated from renal biopsies has not been widely reported; however, renal vein thrombosis may result from thromboembolic events occurring in the renal arcuate veins and cause overt AKI. Etiologic factors that cause this condition are not well understood, and they also happen without predisposing conditions such as nephrotic syndrome (10).

It is essential to treat the underlying precipitant, protect renal function, and prevent complications in RVT. To prevent thrombus progression and emboli, anticoagulation is recommended. Typically, unfractionated Heparin or low-molecular-weight heparin is initiated, followed by Warfarin for 6–12 months or until the underlying nephrotic disease is resolved. There are several indications for thrombectomy and/or thrombolysis in acute RVT, including bilateral RVT, treatment failure while on anticoagulation, thrombosis of the transplanted kidney, and thrombus extension into the inferior vena cava (IVC). It has been shown that fibrinolysis improves renal function and has a low risk of bleeding in the absence of contraindications (11, 12).

Although anticoagulants such as Heparin and Warfarin have been used to treat RVT (13), the effect of direct oral anticoagulants (DOACs) remains to be elucidated. Three patients with RVT treated with direct oral anticoagulants (DOACs) were included in the study conducted by Janczak et al. (14) regarding the use of the DOACs in unusual site VTE. A further four published case reports evaluate Rivaroxaban (15, 16), Apixaban (17), and Edoxaban (18) in patients with RVT with promising clinical outcomes.

Herein we demonstrate a rare case of RAVT, which presented with overt AKI and was treated successfully using Apixaban, a member of the DOACs family.

## Case presentation

A 35 years old woman presented at the emergency department with a three-week history of dull abdominal pain, nausea/vomiting, urinary urgency, and urinary frequency from 2 days after using an Iranian herbal medicine compound for mild upper respiratory symptoms. It was noted that her spouse was positive for COVID-19, 10 days before her gastrointestinal and genitourinary symptoms emerged, and she had also developed mild myalgia and cough concurrent with her husband. On admission, the patient did not appear toxic, had no upper respiratory symptoms, but experienced unremitting abdominal pain. Physical examination was unremarkable except for a high blood pressure of 170/90.

Her medical history was significant for controlled asthma and gastroesophageal reflux disease (GERD), for which she took Salbutamol and Pantoprazole, respectively. There were no indications of alcohol drinking and using illicit substances.

Initial laboratory data showed a white blood cell count of 11.1 × 10^3^/μL; the hemoglobin level was 10.1 g/dL. Her blood urea nitrogen (BUN) and serum creatinine (Cr) levels were 36 mg/dL and 8.53 mg/dL, respectively (Table 1). Hematuria (+1 Hb) was confirmed by urinalysis. However, viral markers, COVID-19 RT-PCR, blood cultures, and pregnancy tests were all negative. High levels of inflammatory markers such as ESR, CRP, and LDH were reported. Furthermore, the 24-h urinalysis did not indicate nephrotic range proteinuria (150 mg/dL in 24 h).

**Table 1:** Laboratory data of the studied case on 1^st^ day, 7^th^ day, and discharge time.

**Blood tests**	**Initial data (on the 1^st^ day, admission time)**	**Pre-dialysis data (on the 7^th^ day, biopsy time)**	**Discharge data**
**Hb**	10.1	11.3	11.6
**WBC**	11.1 × 10^3^/μL	10.8 × 10^3^/μL	10.2 × 10^3^/μL
**Platelets**	232 × 10^3^/μL	240 × 10^3^/μL	228 × 10^3^/μL
**BUN**	36 mg/dL	29 mg/dL	22 mg/dL
**Cr**	8.53 mg/dL	2.92 mg/dL	1.70 mg/dL
**Na** ^**+**^	138	140	142
**K** ^**+**^	4.9	4.5	4.1
**Coombs test direct indirect**	Negative	–	–

Ultrasonographic imaging of the urinary system showed normal size in both kidneys with increased parenchymal echo. A few tiny stones were seen in both kidneys, up to 6 mm in the mid-pole of the left kidney and 3 mm in the mid-pole of the right kidney. Minimal urinary stasis was also seen in the left renal pelvis, with no evidence of hydronephrosis. Color Doppler sonography of both renal vessels was insignificant.

Considering the abnormal findings in renal imaging and laboratory data, the diagnosis of AKI was established, and the patient underwent two episodes of hemodialysis over the next 5 days of admission. However, the second post-dialysis creatinine level stood at 2.92 mg/dL, which was still higher than normal (Table 1).

**Figure 1:**
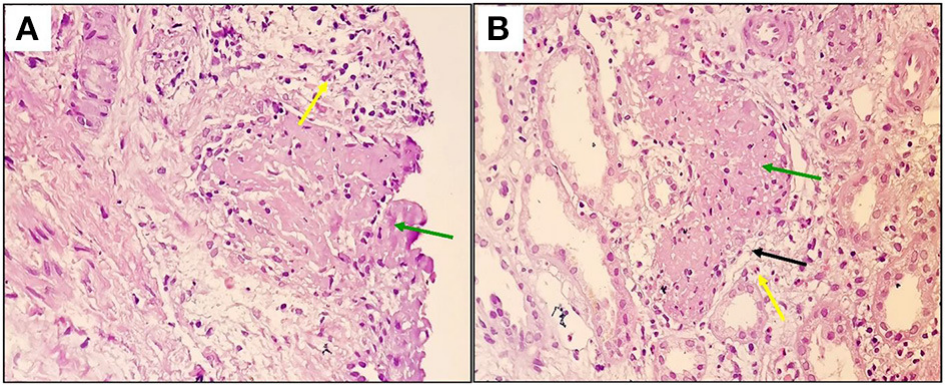
Renal arcuate venous thrombus mainly comprises fibrin and some inflammatory cells [green arrows, **(A, B)**], destruction of the endothelial lining [black arrow, **(B)**], and secondary inflammatory reaction, including fibroblastic proliferation and mononuclear cells [yellow arrows, **(A, B)**] close to the thrombosed vessel can be seen at x200 (Hematoxylin and Eosin staining).

Due to persistent abnormal renal function without a specific etiology, the decision to obtain a renal biopsy was made on the 7^th^ day of admission. Histopathological evaluation of the kidney biopsy indicated RAVT at the corticomedullary junction (Figures 1A, B).

Following the RAVT diagnosis, tests such as immunologic workup, protein S/C deficiency, anti-phospholipid antibody, and factor V Leiden mutation were done, all of which were normal.

Based on the final diagnosis, the treatment decision was made based on discussions in a consulting board consisting of bioethicists, pharmacists, internists, and nephrologists. We decided to initiate oral anticoagulation therapy for this patient. However, we opted against prescribing Warfarin due to the patient's limited access to healthcare facilities for monitoring blood clotting markers. Therefore, she was commenced on Apixaban 10 mg Bid. The patient's serum creatinine level dropped to 1.63 mg/dL after 7 days of DOAC treatment in the hospital. Upon discharge, the Apixaban dosing was adjusted to 5 mg Bid, and a three-month follow-up visit indicated intact renal function with a regular serum creatinine measurement. A follow-up ultrasound scan did not reveal any hydronephrosis or urinary stasis. The patient expressed her satisfaction with the treatment course and did not complain of unwanted side effects.

## Discussion and conclusion

Abnormalities in several elements of the vascular and coagulation systems cause thrombosis. Virchow described thrombosis etiology as the result of irregularities in the vessel wall, platelets, and coagulation proteins. The interaction of these three variables became known as the Virchow triad (19).

RAVT is one of the rarest forms of unusual site venous thromboembolisms (VTEs). To the best of our knowledge, only one previous case series study has demonstrated AKI and RAVT in adults. This study, which evaluated six patients presenting with overt AKI and histopathological findings of RAVT, found that all patients consumed at least moderate amounts of alcohol before showing symptoms (10). However, our case did not report any history of alcohol or illicit substance ingestion.

In the above-mentioned study, several patients also used oral NSAIDs and antibiotics. Overall, it appears that the etiology of renal injury in these cases was due to the ingestion of toxic agents and direct renal injury. In addition, signs of pre-renal etiologies, such as severe dehydration and shock, were absent (10). Similar to these cases, our reported case consumed possibly toxic opium containing traditional herbal medicine compounds. According to the manufacturer, its ingredients include *Zataria multiflora Boiss, Allium Sativum, Heracleum persicum, Satureja hortensis, Dianthus, Foeniculum vulgare*, and trace amounts of opium (20). However, one striking feature of the patient evaluated in our research is the context in which this patient used the medicinal compounds. This medicine was developed for upper respiratory tract symptom relief in COVID-19 patients. Although our workup revealed negative COVID-19 RT-PCR up to 3 times during admission, the patient declared her respiratory symptoms had become evident concomitant with her husband's diagnosis of COVID-19 around 2 days before the onset of her symptoms. False-negative COVID-19 may have also contributed to the presentation of RVT in our case, considering the unexplained high white blood cell count at admission.

Management of acute RVT is based on the presence of AKI. Some resources propose that those concurrently presenting with RVT and AKI should undergo thrombolytic therapy, whereas those without AKI must be treated with therapeutic doses of anticoagulants (21).

Although anticoagulation with Heparin and Warfarin has been studied in RVT patients, the concept of using DOACs in unusual site VTEs is understudied (6, 22). Apixaban has been formerly shown as a reliable drug in treating venous thromboembolism. A randomized clinical trial of 5395 participants concluded that Apixaban therapy did not show inferior results compared to conventional therapy in venous thromboembolism patients (23). One of the few studies regarding DOACs treatment in the setting of VTE in atypical locations demonstrated that there were no statistically significant differences in VTE recurrence and hemorrhage risk between those treated with DOACs such as Rivaroxaban/Apixaban and those conventionally treated with low molecular weight Heparins such as Enoxaparin (14). Other case reports have also shown the effectiveness of Apixaban in setting RVT without acute renal dysfunction (17). In concordance with other studies, our case shows that RVT may be a consequence of COVID-19 infection or even a feature of long COVID. Other types of DOACs, such as Rivaroxaban, have shown positive results in treating RVT in single case studies (16, 24). To the best of our knowledge, our study is the first to have used Apixaban as a treatment for a rare presentation of RVT in the renal arcuate veins.

A patient-centered survey of 200 individuals with VTE indicated that an overwhelming majority of patients chose DOACs over Vitamin K antagonists. Patients expressed the lack of routine laboratory monitoring, the reduced risk of severe hemorrhage, and fewer drug-food interactions as the most compelling reasons to switch to DOACs (25).

Although liver and renal failure are known to be limiting factors in the usage of many DOACs, pharmacokinetic studies have revealed that there is no need for renal adjustment calculations when using Apixaban (26). The renal safety index of Apixaban was also validated in a study on end-stage kidney disease (ESKD) patients with atrial fibrillation (27). Although some guidelines do not favor using DOACs in the setting of renal failure (28), our experience shows that Apixaban, as an exception, is safely tolerated and effective in treating RAVT.

It is important to note that conclusions regarding drug efficacy should only be drawn in randomized clinical trials. Further histopathological studies on patients with toxic substance ingestion and AKI are necessary to elucidate whether or not RAVT is a single and particular entity with identifiable causes.

## Data availability statement

Data reported in this manuscript are available upon reasonable request from the corresponding author.

## Ethics statement

The studies involving human participants were reviewed and approved by the Shiraz University of Medical Sciences. The patients/participants provided their written informed consent to participate in this study. Written informed consent was obtained from the individual(s) for the publication of any potentially identifiable images or data included in this article.

## Author contributions

All authors listed have made a substantial, direct, and intellectual contribution to the work and approved it for publication.
